# Prognostic utility of blood inflammation biomarkers before and after treatment on the survival of patients with locally advanced non‐small cell lung cancer undergoing stereotactic body radiotherapy

**DOI:** 10.1111/crj.13749

**Published:** 2024-04-29

**Authors:** Fang Fang, Zhen Jia, Hongliang Xie, Yangsen Cao, Xiaofei Zhu, Xiao Yu Yang, Xueling Guo, Huojun Zhang

**Affiliations:** ^1^ Department of Radiation Oncology Changhai Hospital Affiliated to Navy Medical University Shanghai China; ^2^ Department of Hepatic Surgery Shanghai Eastern Hepatobiliary Surgery Hospital Shanghai China

**Keywords:** neutrophil–lymphocyte ratio, non‐small cell lung cancer, platelet–lymphocyte ratio, stereotactic body radiation therapy

## Abstract

**Background and Objective:**

The neutrophil–lymphocyte ratio (NLR) and platelet–lymphocyte ratio (PLR) were significant and succinct indicators of systemic inflammation. We assessed the influence of stereotactic body radiotherapy (SBRT) on NLR and PLR in patients with locally advanced non‐small cell lung cancer (LA‐NSCLC).

**Methods:**

We reviewed the medical data of patients with LA‐NSCLC who underwent SBRT between 1 January 2013 and 31 December 2018. NLR and PLR values recorded at pre‐ and post‐SBRT were examined. We assessed the correlation between pre/post‐SBRT NLR and PLR and survival outcomes. The decision tree evaluation was conducted using Chi‐square automatic detection.

**Results:**

In total, 213 patients were included in the study with a median follow‐up duration of 40.00 (ranging from 5.28 to 100.70) months. Upon dichotomization by a median, we identified that post‐SBRT NLR > 5.5 and post‐SBRT PLR > 382.0 were negatively associated with shorter overall survival (OS). In the multivariate assessment, post‐SBRT PLR > 382.0 was the only factor. Based on post‐SBRT PLR, tumor locations, and tumor stage, we categorized patients into low, medium, or high‐risk groups.

**Conclusions:**

Post‐SBRT PLR > 382.0 correlated with survival in patients undergoing SBRT. The decision tree model might play a role in future risk stratification to guide the clinical practice of individualized SBRT for LA‐NSCLC.

## Graphical abstract

The relationship between blood inflammation biomarkers and survival in patients with locally advanced non‐small cell lung cancer undergoing stereotactic body radiotherapy (SBRT) remains unclear. This article presents that post‐SBRT biomarkers correlated with survival, and the decision tree model might play a role in future risk stratification to guide clinical practice.

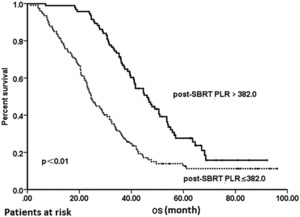

**Abbreviations**
AAPM: American Association of Physicists in Medicine
ACM: all‐cause mortality
ALC: absolute lymphocyte count
BED: biologically equivalent dose
CBC: complete blood count
CI: confidence interval
CRT: chemoradiotherapy
CTV: clinical target volume
DM: distant metastasis
DVHs: dose–volume histograms
GTV: gross target volume
HRs: hazard ratios
LA‐NSCLC: locally advanced non‐small cell lung cancer
LC: local control
LRR: local–regional recurrence
MACEs: major adverse cardiac events
MHD: mean heart dose
NCCN: National Comprehensive Cancer Network
NLR: neutrophil–lymphocyte ratio
OARs: organs at risk
OS: overall survival
PFS: progression‐free survival
PLR: platelet–lymphocyte ratio
PTV: planning target volume
RECIST: Response Evaluation Criteria in Solid Tumors
RIL: radiation‐induced lymphopenia
RT: radiotherapy
SBRT: stereotactic body radiotherapy
SII: systemic immune‐inflammation index

## 1 INTRODUCTION

The unresectable locally advanced non‐small cell lung cancer (LA‐NSCLC) presented significant heterogeneity because of the genetic landscape.^1^ Concurrent chemoradiotherapy (CRT) followed by consolidation immunotherapy is regarded as standard treatment approaches for LA‐NSCLC.^2^, ^3^, ^4^ Nonetheless, a substantial number of patients were not suitable for conventional radiotherapy (RT) with 60 Gy at 2 Gy/Fx for a minimum of 6 weeks. Stereotactic body radiotherapy (SBRT) offers a promising alternative, minimizing irradiation exposure to surrounding normal tissues and circumventing the potential immune suppression observed with expansive treatment fields or more protracted fractionation plans.^5^ Numerous investigations including our prior studies had assessed the effectiveness and safety of SBRT in patients with LA‐NSCLC,^6^, ^7^ with a favorable local control rate of 47.1%–100% and overall survival (OS) of 12–55 months.

Endeavors have been made to shed light on the potential correlation between RT and anti‐tumor immunity. RT may prime the tumor microenvironment by immunostimulatory and immunosuppressive effects.^8^ Compared with conventional multi‐fractionated RT, the more robust anti‐tumor response may be observed by SBRT in a variety of tumors, possibly by endothelial cell damage and activated T cells via released tumor antigens.^9^ Consequently, comprehensive investigations in inflammatory biomarkers during simultaneous CRT might be useful in assessing outcomes in the immunotherapy era, while there was a paucity of reports about it.

It is widely recognized that peripheral blood leukocytes correlate with systemic inflammation, which can be measured using the neutrophils‐lymphocytes ratio (NLR) and platelet‐lymphocyte ratio (PLR).^10^, ^11^ Upon immune system activation, the release of pro‐inflammatory cytokines by immune cells could lead to systemic inflammation. Furthermore, both compromised anti‐tumor immune response and cancer‐related inflammation are believed to be associated with disease progression and prognosis of numerous malignancies.^12^

Increasing studies showed that the NLR and PLR could serve as markers of systemic inflammation. It was demonstrated that elevated NLR and PLR were predictive of unfavorable survival in various cancers.^13^, ^14^ However, there was limited data regarding the use of NLR and PLR to evaluate the response of patients with LA‐NSCLC to SBRT. The aim of this retrospective study was to ascertain if pre‐, post‐treatment, and dynamic changes of NLR and PLR correlate with cancer control and survival in patients with LA‐NSCLC receiving SBRT.

## 2 MATERIALS AND METHODS

### 2.1 Selection of participants

This study was approved by the Institutional Review Board (IRB) of our hospital. Information pertaining to patients, such as staging, pathological findings, therapeutic approaches, and serum laboratory results, were retrieved from the electronic medical records.

Criteria for participation were: (1) ≥18 years with pathological or cytological confirmation of NSCLC; (2) stage III NSCLC based on the 8th edition American Joint Committee on Cancer the tumor, node, and metastasis (TNM) categorization. Clinical staging encompassed CT‐guided biopsy or positron emission tomography (PET) and endobronchial ultrasound (EBUS) and/or mediastinal categorization; (3) patients without surgery or history of thoracic RT; and (4) patients without autoimmune disorders or active infection like acute gastroenteritis, cholecystitis, or appendicitis. Exclusion criteria included: (1) distant metastasis (DM) at diagnosis; (2) a history of second primary cancer; and (3) receiving immunosuppressant or anti‐inflammatory medications prior to treatment. Additionally, regarding central and peripheral lung cancer, tumors invading any important structures within 2 cm of the mediastinum were the central lung cancer. The others were peripheral lung cancers.

### 2.2 SBRT planning and follow‐up

SBRT was delivered using the CyberKnife® system (Accuray Incorporated, Sunnyvale, USA).

CT simulation was conducted with a slice thickness of 1.5 mm. Target delineations were performed by at least two radiation oncologists. Gross target volume (GTV) was defined as a radiographically evident gross disease. A 5 mm margin expansion on GTV formed planning target volume (PTV). However, when tumors were adjacent to the vital organs, the expansion should be omitted in that direction.

The radiation dose of SBRT was determined by the oncologist, partially depending on the tumor's size and position. To account for the variations in dose and fractionation approaches, a biologically equivalent dose (BED) was computed for each patient using an α/β ratio of 10. Task Group 101 (TG‐101), the guideline of dose constraints by the American Association of Physicists in Medicine (AAPM), was referred to assess the dose for organs at risk (OARs). Dosimetric values were derived from dose–volume histograms (DVHs).

Patients were allowed to undergo combined chemotherapy, including induction and adjuvant chemotherapy based on National Comprehensive Cancer Network (NCCN) guidelines. Adjustments of chemotherapy regimens were on the physicians' discretions.

Follow‐ups were performed every 3 months in the first 3 years and every 6 months afterwards. Tumor response was recorded based on the Response Evaluation Criteria in Solid Tumors (RECIST) version 1.1.

### 2.3 Statistical analysis

Laboratory tests were used to determine the complete blood count (CBC). Pre‐SBRT NLR/PLR, calculated by the total neutrophil/platelet count divided by the total lymphocyte count (ALC), was obtained from the latest CBC taken a week before initiating SBRT. Post‐SBRT NLR/PLR was taken from a CBC performed 1 month after treatment. Patients were assigned to two groups based on the median values of NLR and PLR before and after SBRT. Patients' characteristics were analyzed using Pearson's chi‐squared test and Wilcoxon's rank sum test for categorical and numerical data, respectively. The differences between NLR and PLR before and after SBRT were evaluated via paired Wilcoxon's sign rank test. Linear regression analysis was used to identify potential factors associated with NLR and PLR. Factors predictive of OS and PFS were determined by Cox regression analysis.

OS was calculated from the initial date of SBRT to any cause of death or the final follow‐up. The progression‐free survival (PFS) was defined as the time from initiation of SBRT to documentation of any clinical or radiological disease progression or death, whichever occurred first. Local–regional recurrence (LRR) was calculated as the time interval between the administration of SBRT to LRR (recurrence at the site of the primary tumor, the hilar or mediastinal or supraclavicular lymph nodes). The Kaplan–Meier method was utilized to estimate the OS, PFS, and LRR. Log‐rank tests were employed to compare survival between groups. The Cox proportional hazard model was utilized to assess the hazard ratios (HRs) of OS, PFS, and LRR. Multicollinearity between variables was assessed by the variance inflation factor. The OS prognostic model was established using the decision tree evaluation and Chi‐square automatic detection.

Statistical evaluations were conducted using SPSS 25.0. A two‐tailed *p*‐value <0.05 was deemed statistically significant.

## 3 RESULTS

### 3.1 Patient characteristics

We identified records of 311 LA‐NSCLC patients having SBRT. Among these, 47 patients were excluded because of prior surgical resection or RT before SBRT. Twenty patients did not meet the criteria because of incomplete SBRT, while 31 patients did not have both pre‐ and post‐SBRT CBCs. Finally, 213 patients' data were analyzed in this study (Table 1).

**TABLE 1:** Baseline patient, tumor, and treatment characteristics of the overall cohort.

Variable	n	Percentage (%)
Age (yr)		
Md (range)	72 (38‐89)	
Gender		
Male	175	17.8
Female	38	82.2
ECOG		
1	55	25.8
2	158	74.2
History of smoking		
Yes	134	62.9
No	79	37.1
Primary pulmonary diseases		
Yes	85	39.9
No	128	60.1
T stage		
T1	36	16.9
T2	71	33.3
T3	49	23.0
T4	57	26.8
N stage		
N0	12	5.6
N1	22	10.3
N2	102	47.9
N3	77	36.2
TNM stage		
IIIa	108	50.7
IIIb	61	28.6
IIIc	44	20.7
Tumor diameter		
Md (range)	3.8 (1.2‐11.5)	
Pathologic pattern		
Squamous cell carcinoma	93	43.7
Adenocarcinoma	109	51.2
NOS	11	5.2
Primary Tumor Location		
Central	86	40.4
Peripheral	127	59.6
BED_10_		
Md (range)	85.8 (55‐132)	
Type of systemic therapy		
Induction CT+SBRT	177	83.1
Induction CT+SBRT+consolidation CT	17	8.0
Induction TT+SBRT	9	4.2
SBRT alone	10	4.7
Pre‐SBRT NLR		
Md (range)	3.30 (0.63‐16.29)	
Pre‐SBRT PLR		
Md (range)	126.00 (4.38‐700.0)	
Pre‐SBRT lymphocyte Count (K/mL)		
Md (range)	1.57 (0.22‐9.6)	
Post‐SBRT NLR		
Md (range)	5.55 (1.03‐15.21)	
Post‐SBRT PLR		
Md (range)	382.02 (32.42‐1151.92)	
Post‐SBRT Lymphocyte Count (K/mL)		
Md (range)	1.0 (0.12‐2.89)	

Abbreviations: BED_10_, biologically effective dose; CT, chemotherapy; ECOG, Eastern Cooperative Oncology Group; NLR, neutrophil–lymphocyte ratio; NOS, non‐small cell lung cancer not otherwise specified; PLR, platelet–lymphocyte ratio; SBRT, stereotactic body radiotherapy; TT, targeted therapy.

The median follow‐up was 40.00 months (range 5.28–100.70). Overall, 175 were males (82.2%), and 38 were females (17.8%), with an average age of 72 years (range: 38–89 years). A total of 108 (50.7%), 61(28.6%), and 44 (20.7%) patients had stage IIIA, IIIB, and IIIC, respectively. A total of 86 (40.4%) patients had central lung cancer.

The mean radiation dose was 48 Gy ± 6.6 Gy (ranging from 35.0 to 60.0 Gy), and the average fractionation was 6 ± 1.3 Fx (range 5–10 fractions). The median BED_10_ was 85.8 Gy (range: 55–132 Gy). In our study, 177 patients (83.1%) underwent a minimum of 4 cycles of platinum‐based induction chemotherapy as an initial treatment approximately a month prior to SBRT.

### 3.2 Variations in NLR and PLR before and after SBRT

The median pre‐SBRT NLR and PLR were 3.3 (range 0.6–16.3) and 126.0 (range 4.38–700.0), determined 3.5 days before SBRT. At 1 month after SBRT, the median values were 5.5 (range 1.03–15.21) and 382.0 (range 32.4–1151.9) for post‐SBRT NLR and PLR, respectively.

**FIGURE 1:**
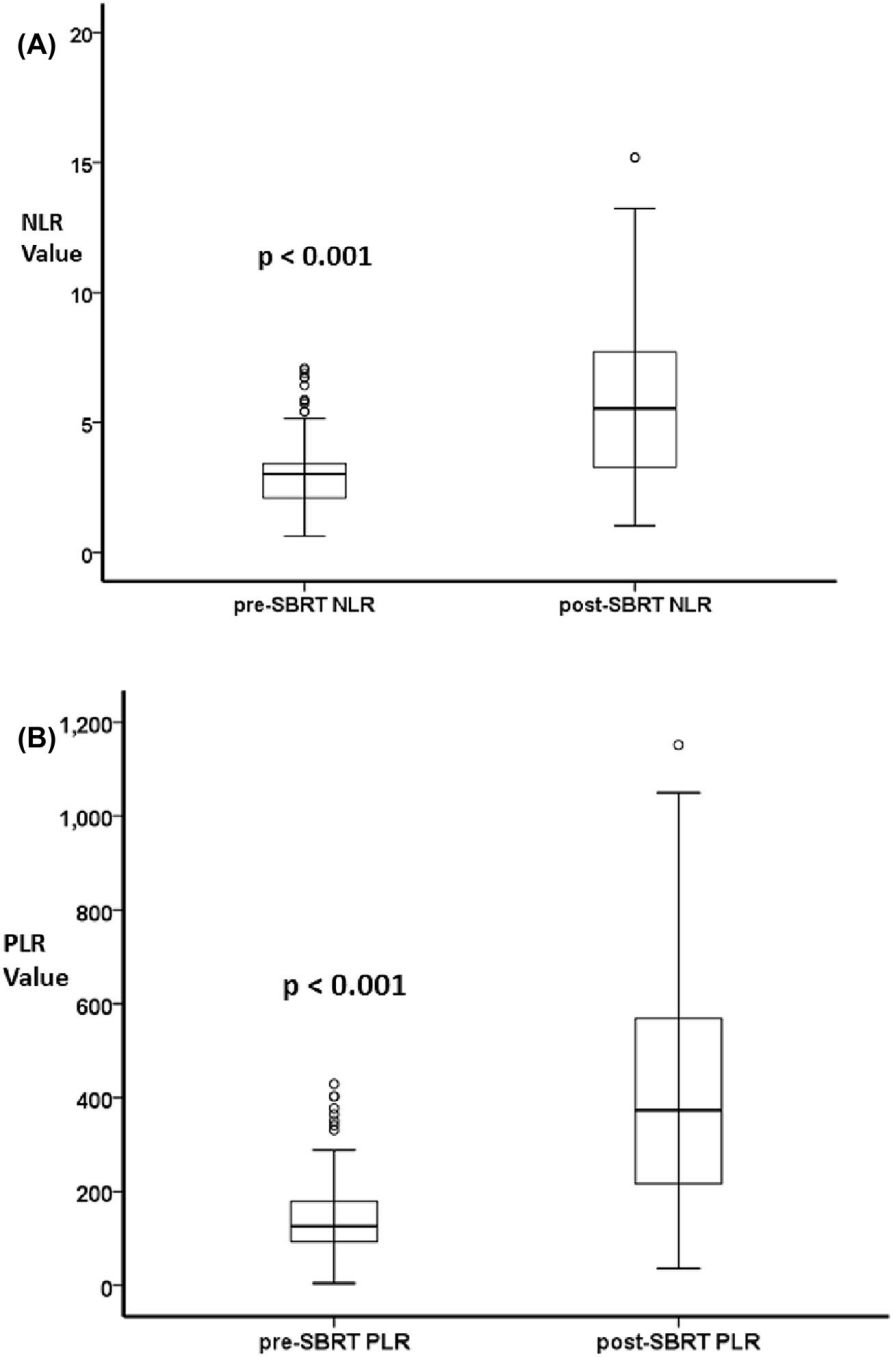
Boxplot of pre‐ and post‐stereotactic body radiotherapy (SBRT) neutrophil–lymphocyte ratio (NLR) (A) and platelet–lymphocyte ratio (PLR) (B) Wilcoxon's signed rank test, *p* < 0.001.

The post‐SBRT NLR increased by a median of 117.45% (range: −79.0% to 851.0%) (*p* < 0.001). The increase was mainly observed in the patients with pre‐SBRT NLR ≤ 3.3, with a median rise of 119.1% (range: 27.9%–226.2%), whereas a median increase of 36.4% (range: −14.4% to 93.4%) was observed in patients with pre‐SBRT NLR > 3.3. The post‐SBRT PLR increased by a median of 171.8% (range: −84.0% to 6105.0%) (*p* < 0.001). Similarly, a significant increase of 287.0% (range: 96.1%–554.3%) was found in pre‐SBRT PLR ≤ 126.0, while a median PLR rise of 119.3% in the case of pre‐SBRT PLR > 126.0 (range: 11.5%–195.4%) (Figure 1).

### 3.3 Factors that influence survival

**FIGURE 2:**
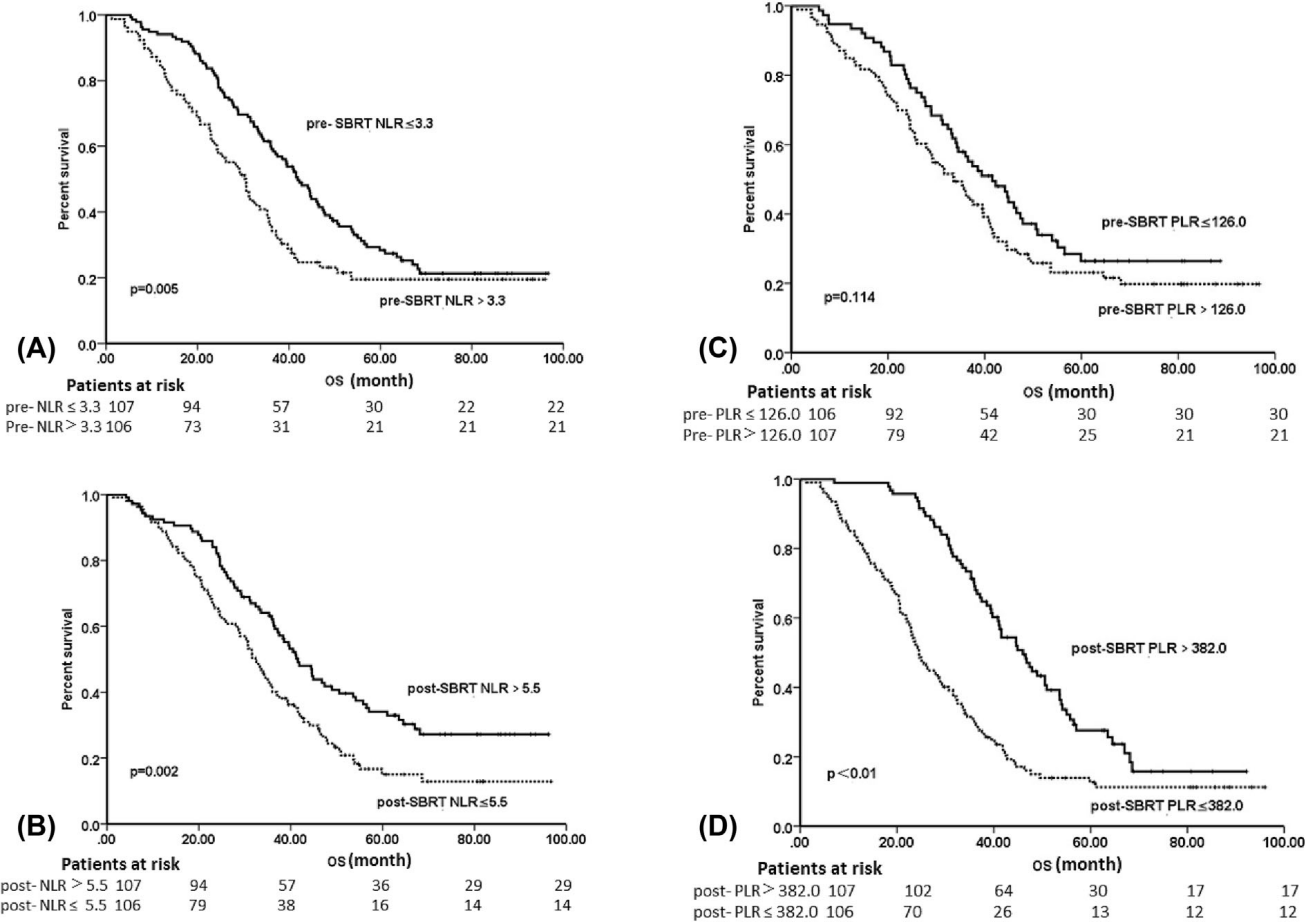
Kaplan–Meier curves for overall survival (OS). (A, B) Patients are stratified by pre‐stereotactic body radiotherapy (SBRT) neutrophil–lymphocyte ratio (NLR) and post‐SBRT NLR. (C, D) Patients are stratified by pre‐SBRT platelet–lymphocyte ratio (PLR) and post‐SBRT NLR.

At the last follow‐up, there were 54 (25.4%) alive. The median OS and PFS were 36.5 months (95% confidence interval [CI], 32.7–40.4 months) and 16.1 months (95% CI, 14.9–17.3 months). The median OS for patients with pre‐SBRT PLR ≤126.0 and >126.0 was 41.9 months (95% CI, 37.6–46.2 months) and 30.3 months (95% CI, 25.0–35.7 months) (*p* = 0.005), respectively. The median PFS for patients with pre‐SBRT PLR ≤126.0 and >126.0 was 17.6 months (95% CI, 15.6–19.6 months) and 13.6 months (95% CI, 10.8–16.4 months) (*p* = 0.040), respectively. The median OS for patients with post‐SBRT PLR ≤382.0 and >382.0 was 53.9 months (95% CI, 45.1–60.7 months) and 24.6 months (95% CI, 22.0–60.7 months) (*p* < 0.001), respectively. The median PFS for patients with post‐SBRT PLR ≤382.0 and >382.0 was 21.6 months (95% CI, 18.2–25.0 months) and 13.8 months (95% CI, 12.7–15.0 months) (*p* < 0.001), respectively. Nevertheless, no significant difference in OS and PFS was found in patients with pre‐SBRT (≤3.3 vs. >3.3) and post‐SBRT NLR (≤5.5 vs. >5.5). Survival curves of different NLR groups and PLR groups are shown in Figure 2.

Predictors of OS and PFS were summarized in Tables 2 and S1. On multivariate analysis, BED_10_ (HR, 1.824; 95% CI, 1.090–3.052; *p* = 0.022), mean heart dose (MHD) V15 (HR, 1.044; 95% CI, 1.005–1.085; *p* = 0.026), and post‐SBRT PLR (HR, 0.233; 95% CI, 0.132–0.413; *p* < 0.001) were predictive of OS. Similarly, on multivariate analysis, BED_10_ (HR, 1.725; 95% CI, 1.082–2.747; *p* = 0.022), primary tumor location (HR, 1.656; 95% CI, 1.024–2.679; *p* = 0.040), and post‐SBRT PLR (HR, 0.375; 95% CI, 0.234–0.601; *p* < 0.001) correlated with PFS.

**TABLE 2:** Univariate and multivariate analysis of factors potentially associated with OS.

Characteristics	Univariate			Multivariate		
	HR	95%CI	P	HR	95%CI	P
Age (yr)	1.002	0.988‐1.017	0.779	NI		
Male vs. female	1.388	0.917‐2.102	0.121	NI		
ECOG	0.991	0.966‐1.017	0.513	NI		
History of smoking (yes)	0.717	0.517‐0.995	0.046	1.327	0.773‐2.277	0.304
Primary pulmonary diseases (yes)	0.943	0.685‐1.300	0.722	NI		
TNM stage	Ref	0.487‐1.080	0.114	NI		
IIIA	0.725	0.543‐1.303	0.438			
IIIB	0.841	0.487‐1.080				
IIIC						
T stage	1.307	1.128‐1.514	<0.001	1.159	0.895‐1.501	0.265
N stage	1.307	1.128‐1.514	0.682	NI		
Pathologic pattern	0.962	0.798‐1.159		NI		
Adenocarcinoma	Ref					
Squamous cell carcinoma	0.524	0.261‐1.050	0.068			
NOS	0.852	0.425‐1.707	0.651			
Primary Tumor Location (Central)	0.535	0.391‐0.732	<0.001	1.124	0.598‐2.113	0.717
BED_10_ (Gy)						
≤85	Ref					
>85	0.625	0.454‐0.860	0.004	1.824	1.090‐3.052	0.022
Heart V15(Gy)	1.077	1.043‐1.113	<0.001	1.044	1.005‐1.085	0.026
Heart max point^a^ dose (Gy)	1.019	1.005‐1.034	0.009	0.986	0.956‐1.016	0.362
Pre‐SBRT NLR(>3.3)	1.579	1.145‐2.178	0.005	0.684	0.352‐1.330	0.263
Pre‐SBRT PLR(>126.0)	1.151	0.807‐1.642	0.438	NI		
Pre‐SBRT ALC (>1.57K/mL)	1.214	0.854‐1.727	0.279	NI		
Post‐SBRT NLR(>5.5)	0.449	0.327‐0.616	<0.001	0.770	0.451‐1.317	0.340
Post‐SBRT PLR(>382.02)	0.222	0.159‐0.311	<0.001	0.233	0.132‐0.413	<0.001
Post‐SBRT ALC (>1.0K/mL)	1.168	0.850‐1.606	0.337	NI		

*Note*: Others includes squamous cell carcinoma and NOS.

Abbreviations: BED10, biologically effective dose; ECOG, Eastern Cooperative Oncology Group; NI, not included in the multivariate model; NLR, neutrophil–lymphocyte ratio; NOS, non‐small cell lung cancer not otherwise specified; OS, overall survival; PLR, platelet–lymphocyte ratio; SBRT, stereotactic body radiotherapy; V15, volume of total heart receiving 15 Gy or more.

^a^ Defined as 0.035 cc or less.

Patterns of tumor failure were evaluated. The dominant type of treatment failure was DM, accounting for 70.9% of all patients (151 of 213 patients). The total rate of local and regional recurrence rate was 34.7% (*n* = 74) and 35.7% (*n* = 76). Factors associated with LRR are shown in Table S2. Post‐SBRT PLR was an independent prognostic factor of LRR (HR, 0.278; 95% CI, 0.696–2.352; *p* < 0.001). A total of 40 (62.6%) and 56 (47.2%) patients with post‐SBRT PLR ≤382.0 and >382.0 had a local failure. The median LRR for post‐SBRT PLR ≤382.0 group and >382.0 group were not reached and 22.6 months, respectively.

### 3.4 Factors associated with post‐SBRT NLR and PLR

Patients were categorized into low or high post‐SBRT NLR and PLR groups based on the median values. Patient characteristics of each cohort were outlined in Table S3. Compared with the lower post‐SBRT NLR cohort, more male patients (*p* = 0.025), older age (*p* = 0.025), higher post‐SBRT NLR (*p* < 0.001), and more central tumors (*p* = 0.001) were observed in patients with higher post‐SBRT NLR. In multivariate linear regression, only the central tumor (β = −1.0149, *p* = 0.021) and post‐SBRT PLR (β = 0.004, *p* < 0.001) were significantly associated with post‐SBRT NLR (Table S4).

Patients demographic and treatment characteristics of each PLR cohort are shown in Table 3. Compared with the lower post‐SBRT PLR cohort, T stages and pathological patterns were different in the higher post‐SBRT PLR cohort, and larger tumor diameter (*p* < 0.001), more central tumors (*p* = 0.004), and higher post‐SBRT NLR (*p* < 0.001) were observed. Yet, when employing stepwise multivariate linear regression, only heart V15 (β = 10.031, *p* = 0.001) and post‐SBRT NLR (β = 12.435, *p* = 0.040) significantly correlated with post‐SBRT PLR (Table S5).

**TABLE 3:** Baseline patient, treatment, and tumor characteristics of the training cohort, stratified by post‐SBRT PLR≤382.0 and > 382.0 groups.

Variable	All patients Percentage (%)	Post‐RT PLR≤367.82 N=107	Post‐RT PLR>367.82 N=106	P value
Post‐RT PLR				
Md (range)	382.02 (32.42‐1151.92)	217.64 (32.42‐382.02)	576.62 (382.52‐1151.92)	<0.001
Age (yr)				
Md (range)	72 (38‐89)	71 (38‐89)	72 (45‐88)	0.386
Gender				
Male	175 (82.2%)	85 (79.4%)	90 (84.9%)	0.297
Female	38 (17.8%)	22 (20.6%)	16 (15.1%)	
History of smoking				
Yes	134 (62.9%)	62 (57.9%)	72 (67.9%)	0.132
No	79 (37.1%)	45 (42.1%)	34 (32.1%)	
Primary pulmonary diseases				
Yes	85 (39.9%)	47 (43.9%)	38 (35.8%)	0.229
No	128 (60.1%)	60 (56.1%)	68 (64.2%)	
T stage				
T1	36 (16.9%)	25 (23.4%)	11 (10.4%)	0.046
T2	71 (33.3%)	37 (34.6%)	34 (32.1%)	
T3	49 (23.0%)	21 (19.6%)	28 (26.4%)	
T4	57 (26.8%)	24 (22.4%)	33 (31.1%)	
N stage				
N0	12 (5.6%)	6 (5.6%)	6 (5.7%)	0.831
N1	22 (10.3%)	9 (8.4%)	13 (12.3%)	
N2	102 (47.9%)	52 (48.6%)	50 (47.2%)	
N3	77 (36.2%)	40 (37.4%)	37 (34.9%)	
TNM stage				
IIIa	108 (50.7%)	54 (50.5%)	54 (50.9%)	0.119
IIIb	61 (28.6%)	36 (33.6%)	25 (23.6%)	
IIIc	44 (20.7%)	17 (15.9%)	27 (25.5%)	
Tumor diameter				
Md (range)	3.8 (1.2‐11.5)	3.30 (1.2‐11.0)	4.2 (1.4‐11.5)	<0.001
Pathologic pattern				
Squamous cell carcinoma	93 (43.7%)	36 (33.6%)	57 (53.8%)	0.004
Adenocarcinoma	109 (51.2%)	67 (62.6%)	42 (39.6%)	
NOS	11 (5.2%)	4 (3.7%)	7 (6.6%)	
Primary Tumor Location				
Central	86 (40.4%)	33 (30.8%)	53 (50.0%)	0.004
Peripheral	127 (59.6%)	74 (69.2%)	53 (50.0%)	
BED_10_				
Md (range)	85.8 (55‐132)	86.4 (59.5‐132.00)	85.5 (52.73‐132.00)	0.314
Type of systemic therapy				
Induction CT+SBRT	177 (83.1%)	88 (83.8%)	89 (82.4%)	0.896
Induction CT+SBRT+consolidation CT	17 (8.0%)	7 (6.7%)	10 (9.3%)	
Induction TT+SBRT	9 (4.2%)	5 (4.8%)	4 (3.7%)	
SBRT alone	10 (4.7%)	5 (4.8%)	5 (4.6%)	
Pre‐SBRT NLR				
Md (range)	3.30 (0.63‐16.29)	2.75 (0.63‐16.29)	3.17 (1.07‐16.14)	0.435
Pre‐SBRT PLR				
Md (range)	126.00 (4.38‐700.0)	123.71 (4.38‐616.59)	130.97 (50.60‐700.0)	1.000
Pre‐SBRT Lymphocyte Count (K/mL)				
Md (range)	1.57 (0.22‐9.6)	1.55 (0.43‐9.60)	1.61 (0.22‐5.05)	0.484
Post‐SBRT NLR				
Md (range)	5.55 (1.03‐15.21)	4.21 (1.03‐15.21)	6.43 (1.19‐15.18)	<0.001
Post‐SBRT Lymphocyte Count (K/mL)				
Md (range)	1.0 (0.12‐2.89)	1.00 (0.23‐2.89)	0.94 (0.12‐2.20)	0.412

*Note*: Others includes squamous cell carcinoma and NOS.

Abbreviations: BED10, biologically effective dose; CT, chemotherapy; NLR, neutrophil–lymphocyte ratio; NOS, non‐small cell lung cancer not otherwise specified; PLR, platelet–lymphocyte ratio; SBRT, stereotactic body radiotherapy; TT, targeted therapy.

### 3.5 Prognostic model for OS

**FIGURE 3:**
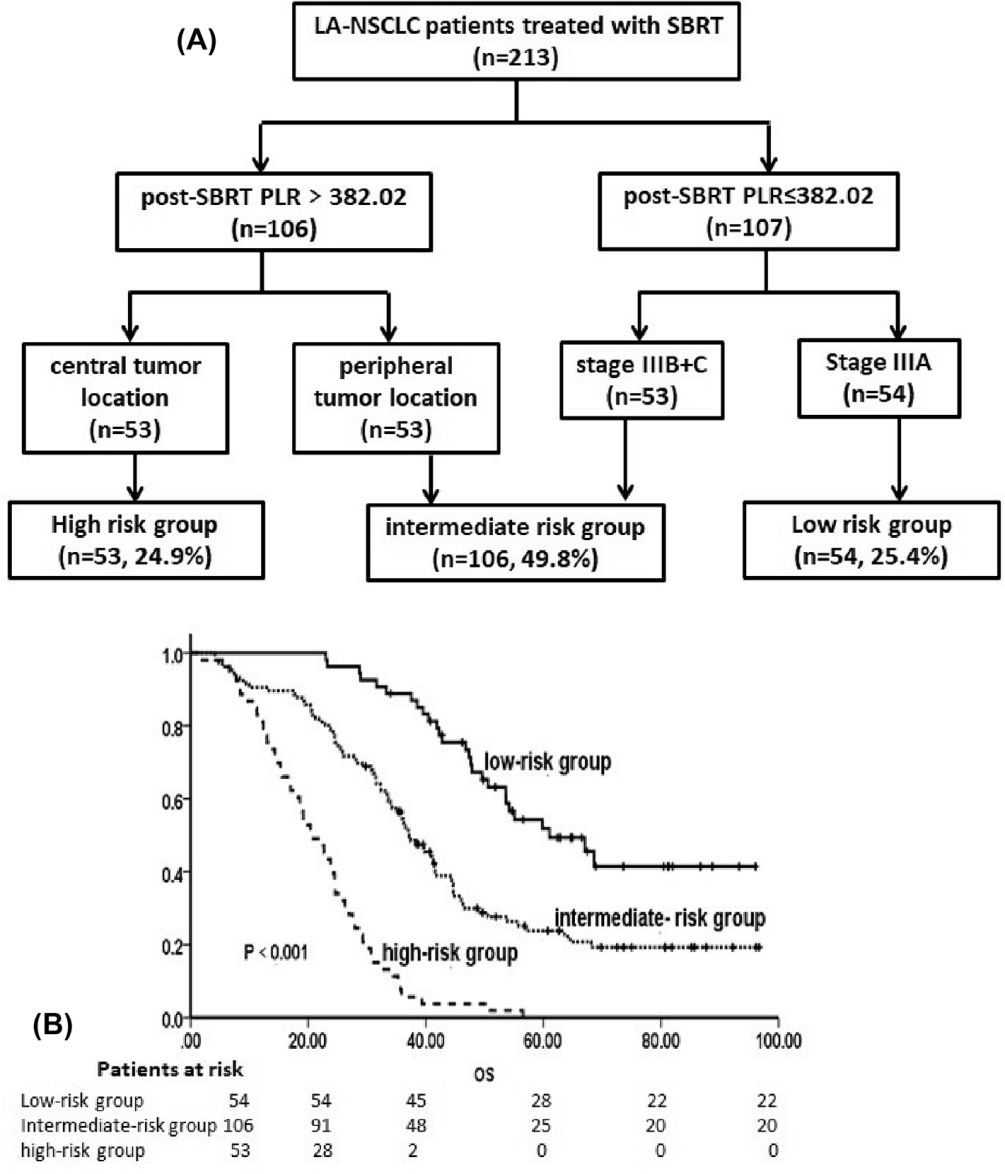
(A) Decision tree analysis results show the prognostic factor. (B) Overall survival (OS) of patients in different risk groups. LA‐NSCLC, locally advanced non‐small cell lung cancer; PLR, platelet‐to‐lymphocyte ratio; SBRT, stereotactic body radiotherapy.

We formulated a prognostic model to estimate OS. On the basis of the decision tree analysis results, post‐SBRT PLR was the primary prognostic factor (Chi‐square = 85.436, *p* < 0.001), followed by tumor location (Chi‐square = 10.952, *p* = 0.001) and tumor stage (Chi‐square = 6.575, *p* = 0.035) (Figure 3). To establish a more clinically pertinent model, groups with similar risk of death were further delineated into three distinct categories: high‐risk group (post‐SBRT PLR > 382.0 with a central tumor), intermediate‐risk group (post‐SBRT PLR > 382.0 with a peripheral tumor or post‐SBRT PLR ≤ 382.0 with stage IIIB or IIIC), and low‐risk group (post‐SBRT PLR ≤ 382.0 with stage IIIA). The 1‐year OS in the high‐, intermediate‐, and low‐risk groups were 81.1%, 90.6%, and 96.3%, respectively, while 3‐year OS were 5.7%, 56.4, and 88.9%, respectively.

## 4 DISCUSSION

The PACIFIC study indicated that adjuvant immunotherapy following definitive chemoradiation significantly improved PFS and OS for LA‐NSCLC.^2^, ^4^ In this study, we evaluated the impact of SBRT on systemic inflammation in LA‐NSCLC patients via inflammatory markers including NLR and PLR. We also examined the correlation between NLR and PLR and outcomes, namely, disease recurrence and OS, in LA‐NSCLC patients. Albeit overstudied NLR or PLR, the difference between previous studies and ours was that we identified that elevated NLR and PLR, namely, dynamic changes of biomarkers, correlated with survival of LA‐NSCLC patients. It was further clarified that post‐SBRT PLR correlated with OS in the current study. Tumor location and tumor stage were also risk factors in the tree decision analysis. Based on this, a novel prognostic model with three distinct risk categories was established for OS. Furthermore, this model might assist physicians in optimizing adjuvant systemic treatment and follow‐up according to the individual patient's risk.

The synergy between immunotherapy and RT is gaining increasing interest.^4^, ^5^, ^15^, ^16^ Radiation‐induced neutrophilia and thrombocytophilia seemed to indicate active inflammation, which facilitated the escape of the immune cells and invasion of the tumor cells.^17^, ^18^, ^19^ Radiation‐induced lymphopenia (RIL) was an immunosuppression factor. One mechanism was inhibiting infiltrations of peripheral blood lymphocytes into the tumor microenvironment and inducing the immunological surveillance escape by weakening the recognition of tumor antigens.^12^ High‐dose RT could lead to increased release of tumor antigens, improved antigen presentation, and enhanced T‐cell infiltration in irradiated tumors.^5^ Data on tumor immune milieu after SBRT were limited. This study tried to establish a model based on predictive biomarkers to estimate the survival of LA‐NSCLC patients with SBRT, which stratified patients into different risk groups to facilitate decision‐making of clinical practice.

More studies investigated a relationship between systemic inflammation and survival in early‐stage NSCLC undergoing SBRT.^18^, ^20^, ^21^, ^22^ Sebastian et al. showed that high pre‐treatment NLR was associated with mortality but not with disease control. Additionally, post‐treatment NLR was significantly increased compared with pre‐treatment one and associated with OS.^21^ Dong et al. found that higher NLR and CRP were associated with worse OS.^18^ Luo et al.^22^ reported that high PLR (≤199.55) was independent prognostic factor of poor survival. A few studies showed the interaction of RT with immune cells and survival in metastatic lung cancer.^23^, ^24^ Chen et al. reported that a reduced absolute lymphocyte count (ALC) is possibly associated with improved response and PFS rates.^23^ In Zhang et al.'s study, the systemic immune‐inflammation index (SII) integrated by immune‐related parameters might be a prognostic factor indicating the risk of recurrence in NSCLC patients with brain metastasis treated with SBRT.^24^

LA‐NSCLC was different from early‐stage and metastatic NSCLC. Considering that the immune status of the patients could predict the efficacy of the consolidation immunotherapy after definitive CRT, it was important to explore the dynamic changes of immune cells after RT as predictors. In line with Kang et al.'s study, it was observed that patients manifesting delayed lymphopenia between 4 and 12 weeks after CRT had shorter OS and PFS. However, no significant survival difference was found between groups stratified by acute lymphopenia.^25^ Another study demonstrated that definitive radiation for LA‐NSCLC could dramatically reduce the ALC and increase the NLR and PLR during RT. Post‐RT NLR and PLR instead of ALC, baseline NLR and PLR were associated with inferior PFS and OS.^26^ Few studies have focused on the application of SBRT in LA‐NSCLC patients. In our study, we found that both NLR and PLR were significantly increased after treatment, and post‐RT PLR value (>382.0) was independent predictors of death. Pre and post‐treatment NLR were associated with survival in univariate analysis, but not in multivariate analysis. While ALC was significantly reduced after treatment, there was no statistical significance between ALC and OS.

There were several studies about the correlation between dose distributions^27^ and doses to specific structures^28^, ^29^ and radiation‐induced dynamic changes in immune cells. Li et al. reported that a low NLR predicted better PFS and OS, and aorta V10 was significantly associated with a high NLR.^29^ In one study, factors such as baseline counts, heart V20, V40, or mean body dose (MBD) were predictors of NLR or PLR 1 month after RT.^26^ In radiation therapy oncology group (RTOG) 0617 it was elucidated that V5, V30, and V40 Gy of the heart correlated with survival.^30^ V50 >25% of the heart was significantly associated with an NLR >10.5 4 months after RT. Keeping V50 of the heart below 25% halved the risk of NLR >10.5.^31^ Multiple studies had highlighted that MHD was associated with all‐cause mortality (ACM) and major adverse cardiac events (MACEs) in patients diagnosed with LA‐NSCLC.^32^, ^33^ Atkins et al. discerned that cardiac substructure particularly coronary artery dose exposure (V15 Gy ≥ 10%) was an independent factor of ACM and MACE.^28^ In our study, T stage, central tumor, MHD, V15 of the heart, and Dmax of the heart significantly correlated with post‐RT PLR. In stepwise multivariate linear regression, V15 of the heart was associated with post‐SBRT PLR. SBRT may minimize the heart dose and body dose, which might reduce the adverse effect of radiation on NLR or PLR. Before our study, none had tried to estimate OS by the decision tree model in LA‐NSCLC patients receiving SBRT. Based on PLR, tumor location, and tumor stage, we classified patients into low, intermediate, or high‐risk groups. The decision tree model may have a future role in risk stratification to inform the individualized delivery of SBRT to LA‐NSCLC.

There were several limitations in our study. First of all, due to the retrospective nature, the systemic chemotherapy regimens were heterogeneous, which may affect survival. Additionally, though we included potential factors in the multivariate analyses to identify the predictors of outcomes, there were still confounding factors that may be not considered. Therefore, interpretations of the results should be cautious. Secondly, serum inflammatory indicators alone may not be sufficient to demonstrate the correlation between anti‐tumor immune response induced by SBRT and survival. Genetic and immune profiling may be further investigated. Moreover, well‐designed prospective studies are required to clarify the effects of SBRT on anti‐tumor immunity and survival with those biomarkers.

## 5 CONCLUSIONS

Our study tried to identify the potential biomarkers to predict changes in the host immune status of patients receiving SBRT. In summary, we observed post‐SBRT PLR value to be independently associated with OS in patients with unresectable LA‐NSCLC who underwent SBRT. The tree decision analysis was also validated to be a novel prognostic model for OS, which may be outperforming PLR. These clinical indicators may guide the combination of immunotherapy and RT for the treatment of LA‐NSCLC. Further studies are needed to validate that SBRT affects those patients' immunologic states.

## AUTHOR CONTRIBUTIONS

All authors contributed to the study's conception and design. Material preparation, data collection, and analysis were performed by Fang Fang, Zhen Jia, Hongliang Xie, Yangsen Cao, Xiaofei Zhu, XiaoYu Yang, Xueling Guo, and Huojun Zhang. The first draft of the manuscript was written by Fang Fang and all authors commented on previous versions of the manuscript. All authors contributed to the article and approved the submitted version.

## CONFLICT OF INTEREST STATEMENT

The authors declare that the research was conducted in the absence of any commercial or financial relationships that could be construed as a potential conflict of interest.

## ETHICS STATEMENT

The study was conducted according to the guidelines of the Declaration of Helsinki, and approved by the local Ethics Committee of Medical Faculty, Changhai Hospital Affiliated to Navy Medical University. Patient consent was waived as the data were only analyzed in an anonymized form.

## Supporting information


**Table S1.** Treatment‐related toxicities for 213 patients with LA‐NSCLC by SBRT [n (%)].

## Data Availability

The data that support the findings of this study are available from the corresponding author upon reasonable request.
