# Supplementary material for: Prognostic utility of blood inflammation biomarkers before and after treatment on the survival of patients with locally advanced non‐small cell lung cancer undergoing stereotactic body radiotherapy
Source: Clin Respir J. 2024 Apr 29;18(5):e13749. doi: 10.1111/crj.13749 (PMC11058398; doi:10.1111/crj.13749)
Supplement: Supplementary file 1 — Table S1. Treatment‐related toxicities for 213 patients with LA‐NSCLC by SBRT [n (%)]. [file CRJ-18-e13749-s001.docx]

**Supplementary table 1** Univariate and multivariate analysis of factors potentially associated with PFS.

| Characteristics | Univariate | | | Multivariate | | |
| --- | --- | --- | --- | --- | --- | --- |
|  | HR | 95%CI | P | HR | 95%CI | P |
| Age (yr) | 0.995 | 0.983-1.008 | 0.475 | NI |  |  |
| Male vs. female | 1.236 | 0.856-1.785 | 0.258 | NI |  |  |
| KPS | 0.997 | 0.974-1.020 | 0.775 | NI |  |  |
| History of smoking(yes) | 1.332 | 0.991-1.789 | 0.057 | NI |  |  |
| Primary pulmonary diseases(yes) | 0.846 | 0.631-1.133 | 0.262 | NI |  |  |
| TNM stage |  |  |  |  |  |  |
| IIIA | Ref |  |  |  |  | 0.187 |
| IIIB | 0.595 | 0.412-0.859 | 0.006 | 0.745 | 0.539-1.030 | 0.074 |
| IIIC | 0.822 | 0.554-1.218 | 0.328 | 1.214 | 0.879-1.677 | 0.238 |
| T stage | 1.268 | 1.109-1.450 | 0.001 | 1.116 | 0.873-1.427 | 0.380 |
| N stage | 1.082 | 0.898-1.303 | 0.409 | NI |  |  |
| Pathologic pattern |  |  |  | NI |  |  |
| Adenocarcinoma | Ref |  |  |  |  |  |
| Squamous cell carcinoma | 0.718 | 0.374-1.377 | 0.319 |  |  |  |
| NOS | 0.925 | 0.479-1.786 | 0.816 |  |  |  |
| Primary Tumor Location (Central) | 0.657 | 0.492-0.877 | 0.004 | 1.656 | 1.024-2.679 | 0.040 |
| BED_10_ (Gy) |  |  |  |  |  |  |
| ≤85 | Ref |  |  |  |  |  |
| ＞85 | 1.599 | 1.192-2.144 | 0.002 | 1.725 | 1.082-2.747 | 0.022 |
| Mean heart dose V15(Gy) | 1.068 | 1.035-1.101 | ＜0.001 | 1.002 | 0.934-1.076 | 0.950 |
| Heart max point** dose(Gy) | 1.015 | 1.001-1.029 | 0.034 | 1.013 | 0.993-1.033 | 0.218 |
| Pre-SBRT NLR(＞3.3) | 1.361 | 1.013-1.828 | 0.041 | 0.767 | 0.431-1.365 | 0.367 |
| Pre-SBRT PLR(＞126.0) | 1.388 | 1.006-1.915 | 0.046 | 1.088 | 0.599-1.978 | 0.781 |
| Pre-SBRT ALC (＞1.57K/mL) | 1.067 | 0.777-1.466 | 0.688 | NI |  |  |
| Post-SBRT NLR(＞5.55) | 1.309 | 0.985-1.740 | 0.064 | 1.523 | 0.963-2.409 | 0.072 |
| Post-SBRT PLR(＞382.02) | 2.298 | 1.710-3.087 | ＜0.001 | 0.375 | 0.234-0.601 | ＜0.001 |
| Post-SBRT ALC (＞1.0K/mL) | 1.132 | 0.844-1.517 | 0.409 | NI |  |  |

PFS, progression-free survival; NI, not included in the multivariate model; *Others includes squamous cell carcinoma and NOS; ** defined as 0.035cc or less.

**Supplementary table 2** Univariate and multivariate analysis of factors potentially associated with LRR

| Characteristics | Univariate | | | Multivariate | | |
| --- | --- | --- | --- | --- | --- | --- |
|  | HR | 95%CI | P | HR | 95%CI | P |
| Age (yr) | 1.010 | 0.991-1.029 | 0.314 | NI |  |  |
| Male vs. female | 1.206 | 0.714-2.038 | 0.484 | NI |  |  |
| KPS | 0.998 | 0.966-1.032 | 0.919 | NI |  |  |
| History of smoking(yes) | 1.250 | 0.825-1.895 | 0.293 | NI |  |  |
| Primary pulmonary diseases(yes) | 0.763 | 0.501-1.163 | 0.209 | NI |  |  |
| TNM stage |  |  |  |  |  |  |
| IIIA | Ref |  |  |  |  |  |
| IIIB | 0.711 | 0.542-0.932 | 0.014 | 0.691 | 0.324-1.474 | 0.340 |
| IIIC | 1.091 | 0.816-1.458 | 0.558 | 1.225 | 0.524-2.861 | 0.639 |
| T stage | 1.381 | 1.141-1.670 | 0.001 | 1.028 | 0.734-1.438 | 0.874 |
| N stage | 1.073 | 0.836-1.376 | 0.581 | NI |  |  |
| Pathologic pattern |  |  |  | NI |  |  |
| Adenocarcinoma | Ref |  |  | NI |  |  |
| Squamous cell carcinoma | 0.510 | 0.230-1.133 | 0.098 | NI |  |  |
| NOS | 0.754 | 0.339-1.678 | 0.489 | NI |  |  |
| Primary Tumor Location (Central) | 0.473 | 0.316-0.707 | ＜0.001 | 1.100 | 0.549-2.206 | 0.788 |
| BED_10_ (Gy) |  |  |  |  |  |  |
| ≤85 | Ref |  |  |  |  |  |
| ＞85 | 6.187 | 3.991-9.591 | ＜0.001 | 7.312 | 3.634-14.715 | ＜0.001 |
| Mean heart dose V15(Gy) | 1.074 | 1.034-1.116 | ＜0.001 | 1.028 | 0.977-1.082 | 0.289 |
| Heart max point** dose(Gy) | 1.013 | 0.996-1.031 | 0.143 | NI |  |  |
| Pre-SBRT NLR(＞3.3) | 0.637 | 0.423-0.958 | 0.030 | 0.761 | 0.391-1.482 | 0.422 |
| Pre-SBRT PLR(＞126.0) | 1.383 | 0.867-2.207 | 0.174 | NI |  |  |
| Pre-SBRT ALC (＞1.57K/mL) | 1.197 | 0.758-1.891 | 0.440 | NI |  |  |
| Post-SBRT NLR(＞5.5) | 1.428 | 0.955-2.137 | 0.083 | 1.280 | 0.696-2.352 | 0.427 |
| Post-SBRT PLR(＞382.0) | 2.233 | 1.478-3.375 | ＜0.001 | 0.278 | 0.696-2.352 | ＜0.001 |
| Post-SBRT ALC (＞1.0K/mL) | 0.859 | 0.568-1.299 | 0.470 | NI |  |  |

LRR, Local-regional recurrence; NI, not included in the multivariate model; *Others includes squamous cell carcinoma and NOS; ** defined as 0.035cc or less.

**Supplementary table 3** Baseline patient, treatment, and tumor characteristics of the training cohort, stratified by post-SBRT NLR≤5.55 and > 5.55 groups.*

| Variable | All patients  Percentage (%) | Post-SBRT NLR≤5.55  N=105 | Post-SBRT NLR＞5.55  N=108 | P value |
| --- | --- | --- | --- | --- |
| Post-RT NLR |  |  |  |  |
| Md (range) | 5.55(1.03-15.21) | 3.26(1.03-5.50) | 7.71(5.51-15.21) | < 0.001 |
| Age (yr) |  |  |  |  |
| Md (range) | 72(38-89) | 70(41-89) | 74(38-88) | 0.046 |
| Gender |  |  |  |  |
| Male | 175(82.2%) | 80(76.2%) | 95(88.0%) | 0.025 |
| Female | 38(17.8%) | 25(23.8%) | 13(12.0%) |  |
| KPS |  |  |  |  |
| 90 | 71(33.3%) | 35(33.3%) | 36(33.3%) | 0.981 |
| 80 | 125(58.7%) | 62(59.0%) | 63(58.3%) |  |
| 70 | 17(8.0%) | 8(7.6%) | 9(8.3%) |  |
| History of smoking |  |  |  |  |
| Yes | 134(62.9%) | 60(57.1%) | 74(68.5%) | 0.086 |
| No | 79(37.1%) | 45(42.9%) | 34(31.5%) |  |
| Primary pulmonary diseases |  |  |  |  |
| Yes | 85(39.9%) | 41(39.0%) | 44(40.7%) | 0.801 |
| No | 128(60.1%) | 64(61.0%) | 64(59.3%) |  |
| T stage |  |  |  |  |
| T1 | 36(16.9%) | 15(14.3%) | 21(19.4%) | 0.245 |
| T2 | 71(33.3%) | 37(35.2%) | 34(31.5%) |  |
| T3 | 49(23.0%) | 29(27.6%) | 20(18.5%) |  |
| T4 | 57(26.8%) | 24(22.9%) | 33(30.6%) |  |
| N stage |  |  |  |  |
| N0 | 12(5.6%) | 15(14.3%) | 21(19.4%) | 0.245 |
| N1 | 22(10.3%) | 37(35.2%) | 34(31.5%) |  |
| N2 | 102(47.9%) | 29(27.6%) | 20(18.5%) |  |
| N3 | 77(36.2%) | 24(22.9%) | 33(30.6%) |  |
| TNM stage |  |  |  |  |
| IIIa | 108(50.7%) | 50(47.6%) | 58(53.7%) | 0.628 |
| IIIb | 61(28.6%) | 31(29.5%) | 30(27.8%) |  |
| IIIc | 44(20.7%) | 24(22.9%) | 20(18.5%) |  |
| Tumor diameter |  |  |  |  |
| Md (range) | 3.8(1.2-11.5) | 3.7(1.2-11.5) | 4.0(1.4-11.0) | 0.304 |
| Pathologic pattern |  |  |  |  |
| Squamous cell carcinoma | 93(43.7%) | 38(36.2%) | 55(50.9%) | 0.073 |
| Adenocarcinoma | 109(51.2%) | 62(59.0%) | 47(43.5%) |  |
| NOS | 11(5.2%) | 5(4.8%) | 6(5.6%) |  |
| Primary Tumor Location |  |  |  |  |
| Central | 86(40.4%) | 31(29.5%) | 55(50.9%) | 0.001 |
| Peripheral | 127(59.6%) | 74(70.5%) | 53(49.1%) |  |
| BED_10_ |  |  |  |  |
| Md (range) | 85.8(55-132) | 86.4(59.5-132.00) | 85.5(52.73-132.00) | 0.314 |
| Type of systemic therapy |  |  |  |  |
| Induction CT＋SBRT | 177(83.1%) | 87(82.9%) | 90(83.3%) | 0.981 |
| Induction CT＋SBRT＋consolidation CT | 17(8.0%) | 9(8.6%) | 8(7.4%) |  |
| Induction TT＋SBRT | 9(4.2%) | 4(3.8%) | 5(4.6%) |  |
| SBRT alone | 10(4.7%) | 5(4.8%) | 5(4.6%) |  |
| Pre-SBRT NLR |  |  |  |  |
| Md (range) | 3.30(0.63-16.29) | 2.92(0.63-12.50) | 3.10(1.07-16.29) | 0.073 |
| Pre-SBRT PLR |  |  |  |  |
| Md (range) | 126.00(4.38-700.0) | 125.95(4.38-616.59) | 126.48(52.91-700.0) | 0.857 |
| Pre-SBRT Lymphocyte Count (K/mL) |  |  |  |  |
| Md (range) | 1.57(0.22-9.6) | 1.57(0.43-9.60) | 1.60(0.22-3.33) | 0.940 |
| Post-SBRT PLR |  |  |  |  |
| Md (range) | 382.0(32.42-1151.92) | 294.5(21.42-943.58) | 478.1(39.31-1151.92) | ＜0.001 |
| Post-SBRT Lymphocyte Count (K/mL) |  |  |  |  |
| Md (range) | 1.0(0.12-2.89) | 0.95(0.12-2.89) | 1.00(0.13-2.27) | 0.414 |

**Supplementary table 4** Univariate and multivariate linear regression associating variables with post-SBRT NLR

| Characteristics | Univariate | | | Multivariate | | |
| --- | --- | --- | --- | --- | --- | --- |
|  | β | 95%CI | P | β | 95%CI | P |
| Age (yr) | 0.049 | 0.011-0.086 | 0.012 |  |  | 0.063 |
| Male vs. female | 0.661 | -0.398-1.720 | 0.220 |  |  |  |
| KPS | 0.010 | -0.059-0.079 | 0.775 |  |  |  |
| History of smoking(yes) | 0.380 | -0.461-1.221 | 0.374 |  |  |  |
| T stage | 0.113 | -0.272-0.498 | 0.563 |  |  |  |
| Primary Tumor Location (Central) | -1.220 | -2.033—0.408 | 0.003 | -1.0149 | -1.940--0.157 | 0.021 |
| BED_10_ (Gy) | -0.003 | -0.025-0.020 | 0.812 |  |  |  |
| Mean heart dose V15(Gy) | -0.001 | -0.090-0.087 | 0.975 |  |  |  |
| Heart max point** dose(Gy) | 0.010 | -0.031-0.052 | 0.625 |  |  |  |
| Pre-SBRT NLR | 0.167 | -0.017-0.350 | 0.074 |  |  | 0.152 |
| Pre-SBRT PLR | 0.001 | -0.004-0.006 | 0.616 |  |  |  |
| Pre-SBRT ALC | -0.438 | -0.947-0.071 | 0.091 |  |  |  |
| Post-SBRT PLR | 0.004 | 0.003-0.006 | ﹤0.001 | 0.004 | 0.002-0.006 | ﹤0.001 |
| Post-SBRT ALC | -0.704 | -1.538-0.129 | 0.097 |  |  |  |

**Supplementary table 5** Univariate and multivariate linear regression associating variables with post-SBRT PLR

| Characteristics | Univariate | | | Multivariate | | |
| --- | --- | --- | --- | --- | --- | --- |
|  | β | 95%CI | P | β | 95%CI | P |
| Age (yr) | 1.034 | -1.904-3.972 | 0.489 |  |  |  |
| Male vs. female | 37.511 | -43.932-118.954 | 0.365 |  |  |  |
| KPS | 1.468 | -3.818-6.753 | 0.585 |  |  |  |
| History of smoking(yes) | 41.754 | -22.673-106.182 | 0.203 |  |  |  |
| T stage | 47.158 | 18.258-76.058 | 0.002 |  |  | 0.070 |
| Primary Tumor Location (Central) | -79.543 | -142.295--16.791 | 0.013 |  |  | 0.224 |
| BED_10_ (Gy) | -0.873 | -2.577-0.831 | 0.314 |  |  |  |
| Mean heart dose V15(Gy) | 10.258 | 4.633-15.882 | ﹤0.001 | 10.031 | 4.488-15.573 | 0.001 |
| Heart max point** dose(Gy) | 3.805 | 0.774-6.835 | 0.014 |  |  | 0.079 |
| Pre-SBRT NLR | 4.895 | -9.279-19.069 | 0.497 |  |  |  |
| Pre-SBRT PLR | 0.024 | -0.042-0.089 | 0.473 |  |  |  |
| Pre-SBRT ALC | -5.604 | -43.761-32.553 | 0.772 |  |  |  |
| Post-SBRT NLR | 15.444 | 15.612-35.275 | ﹤0.001 | 12.318 | 0.184-24.452 | 0.047 |
| Post-SBRT ALC | -34.861 | -98.370-28.648 | 0.280 |  |  |  |
